# Do important points change the game? Technical-tactical performance in high-level men’s padel

**DOI:** 10.5114/biolsport.2026.158680

**Published:** 2026-04-20

**Authors:** Rafael Conde-Ripoll, Adrián Escudero-Tena, Jesús Ramón-Llin, Álvaro Bustamante-Sánchez

**Affiliations:** 1 Universidad Europea de Madrid. Department of Sports Sciences. Faculty of Medicine, Health and Sports, Madrid, Spain; 2 Research Group on Sports Technique and Tactics, University of Valencia, 46010 Valencia, Spain

**Keywords:** Pressure, Tiebreak, Break point, Golden point, Match analysis, Systematic observation

## Abstract

In padel, match outcomes often hinge on high-pressure situations that arise during critical scoring moments. However, little is known about how technical–tactical performance varies between important and regular points. This study aimed to test whether important points (break, golden, tiebreak) differ from regular points (all others) in last-shot effectiveness, server success, and set-winner success in high-level men’s padel, and to examine shot-family distributions by point importance. Through systematic observation, data were collected from 38 matches (4,521 points). Outcomes were winner (W), forced error (FE), unforced error (UE), serving pair wins the point (SrvWin), and point won by the set-winning pair (SetWinPt). Binomial GLMMs (logit; random intercept for match) were fitted; binomial GLMs were used when the random-effect variance was at the boundary. We contrasted break, golden, and tiebreak points with regular and reported odds ratios (OR, 95% CI) and model-adjusted probabilities. Shot-family distributions for the last shot (W/FE/UE) were compared with χ^2^ tests. Important versus regular points showed no meaningful differences (ORs ≈ 1.00); model-adjusted probability gaps were ≤ 2.3 percentage points. By point type, omnibus tests were non-significant and no pairwise contrast versus regular survived Holm adjustment. Shot-family composition did not differ between important and regular points for W, FE, or UE. In high-level men’s padel, point importance did not systematically affect last-shot effectiveness, server or set-winner success, or final-shot family. These findings suggest performance stability under pressure. Therefore, coaches may prioritize consolidating consistent execution across contexts using pressure drills to reinforce existing routines.

## INTRODUCTION

Performance analysis in sport aims to derive objective information, typically operationalised as key performance indicators (KPIs), from athletes’ behaviours in real competitive contexts [1, 2]. This process provides coaches and players with applied knowledge that supports evidence-based decision-making and facilitates the systematic enhancement of performance [3–6]. In padel, most investigations have centred on this dimension within professional players [7]. However, performance analysis in high-level or semi-professional padel remains largely unexplored.

In padel, match outcomes are often determined by high-pressure situations that emerge during critical scoring scenarios [8–10]. Break points, golden points, and tiebreaks represent particularly decisive moments, as they can directly shift the balance of a set or match. To secure a set, at least one service break is required, typically achieved through winning a break point or a golden point, unless the score reaches 6–6, which leads to a set tiebreak [11]. In this case, an additional mini-tiebreak must be contested to resolve the set. Furthermore, in certain tournaments the final set is not played as a conventional set but rather as a super tiebreak (also referred to as a match tiebreak), in which the first pair to reach 10 points is declared the winner [11].

Research in racquet sports indicates that high-stakes points impose unique psychological and tactical demands. Because of their decisive nature, these important points carry disproportionately greater importance than regular play and often elicit performance shifts under pressure [12–14]. Two contrasting responses are typically observed: choking, characterised by performance deterioration under stress due to heightened self-consciousness, disrupted automaticity, or anxiety-driven attentional shifts [15–17], and clutch performance, the capacity to raise execution levels in critical moments through increased concentration, self-confidence, and adaptive coping strategies [16–18]. In padel, important points encompass both possibilities, offering a valuable framework to investigate how competitive pressure shapes technical–tactical behaviour in high-level play.

Even though research comparing important vs. regular points exists in other racquet sports like tennis [12, 14, 19, 20], to the best of our knowledge, just one study has focused on comparing key moments, golden points and non-key moments in professional padel [21], revealing that in men’s professional padel, as the importance of the point increases (non-key moment < key moment < golden point), the ratio error: winner is higher.

Despite these insights, little is known about how technical-tactical performance indicators differ between important and regular points in padel. Therefore, this study investigated whether technical-tactical performance at the point level differs between important points—defined as break points, golden points, and tiebreak points—and regular points in high-level men’s padel. Our primary aim was to compare the probabilities of (i) last-shot effectiveness—winner (W), forced error (FE), unforced error (UE)—(ii) the serving pair winning the point, and (iii) the set-winning pair winning the point between important and regular points; we also contrasted each important-point context (break, golden, tiebreak) separately with regular points. As a secondary aim, we described whether the distribution of last-shot families (shot-type groups) differed between important and regular points within W, FE, and UE. We hypothesised that: (i) the probability of making a winner, a forced error, an unforced error, the serving pair winning the point, and the set-winning pair winning the point would differ between regular and important points, with differences potentially varying across break, golden, and tiebreak contexts; (ii) technical-tactical patterns would also differ by context, with a higher proportion of unforced errors occurring on smashes, *bandejas* and returns during important points compared to regular points, and a higher proportion of winners on volleys during regular points compared to important points. By examining all this, the findings seek to deepen our understanding of pressure performance in high-level men’s padel and provide practical applications for training design, match preparation, and in-match decision-making.

## MATERIALS AND METHODS

### Research design

This observational study follows an empirical methodology with a descriptive approach, characterized by a nomothetic, punctual, and multidimensional framework [22], meaning that it included plurality of observation units (nomothetic), comprised observations without longitudinal follow-up (punctual), and analyzed multiple dimensions of behavior simultaneously (multidimensional).

### Sample

A total of 4,521 points were analyzed from 38 matches, including 18 pressure training matches and 20 official competition matches. We decided to include both kind of matches since previous research has shown that training under pressure mirrors competition in highlevel male padel players from a technical-tactical performance standpoint [23]. Out of these 4,521 points, 647 were considered important points (104 were tiebreak points, 199 were golden points, and 344 were break points) and the rest were considered regular points. The matches took place in Finland during 2022 and 2023. All competition matches were part of tournaments in the highest category, with points contributing to the Finnish Federation ranking.

Participants were recruited using a convenience sampling approach, selecting players who were actively competing at the highest level in Finland. The sample comprised 30 male players, all ranked within the top 60 of the Finnish Federation at the time of data collection. In the pressure training matches, 12 players from the top 25 participated. In the official competition matches, these 12 players were joined by an additional 18 players, all ranked within the top 60. Although not all players participated in both training and competition scenarios, this ranking distribution ensures that the level of play was consistently high across all analyzed matches.

Players were excluded if they were injured or unable to participate in either the training or competition matches during the study period. No additional exclusion criteria were applied.

The sample size was not determined using an a priori power calculation due to the recruitment approach. Instead, the study included all available data from matches and training sessions during the study period, providing a comprehensive and robust dataset for analysis. All procedures were conducted according with ethical standards in sport and exercise science research [24] and in accordance with the Helsinki Declaration. The research was previously approved by the Ethics Committee for Research of the Universidad Europea de Madrid with the code CIPI/22.303.

### Study variables

#### Unit of analysis

The unit of analysis was the point. Each point was identified by match and set number.

#### Point importance (exposures)

Point importance was coded using three binary indicators (0 = no, 1 = yes): break point (the receiving pair could win the game), golden point (deciding point at 40–40), and tiebreak point (points played within a set tiebreak or super tiebreak). From these, a composite important point variable was created (1 if any of the above were present; otherwise 0). For secondary contrasts, a four-level point type factor was defined with levels Regular, Break, Golden, and Tiebreak; in those analyses, Regular denotes points that were not break, not golden, and not tiebreak.

#### Last shot effectiveness

It was classified as Winner (W), Forced error (FE), or Unforced error (UE). For modelling and descriptive purposes, we additionally derived binary indicators (0/1) for each category (e.g., UE = 1 if the last-shot effectiveness was Unforced error, else 0). Two further point-level outcomes were coded: serving pair wins the point (1 = serving pair won, 0 = returning pair won) and point won by the eventual set winners (1 = point won by the pair who won the set, 0 = set-losing pair).

#### Point outcome depending on the serve situation, and point outcome depending on the serve situation

A difference was made between serving and returning pair. A difference was made between set-winning and set-losing pair.

That’s why two further point-level outcomes were coded: serving pair wins the point (1 = serving pair won, 0 = returning pair won) and point won by the eventual set winners (1 = point won by the pair who won the set, 0 = set-losing pair).

#### Shot descriptors

The last shot type was recorded using technical categories (e.g., smash, *bandeja*, forehand/backhand volley, forehand/backhand groundstroke, back wall forehand/backhand, etc.). To avoid sparse categories, these were consolidated a priori into shot-type families: Smash, *Bandeja*, Volleys (forehand/backhand volley), Groundstrokes (forehand/backhand), Walls, and Other (Table 1).

**TABLE 1:** List of shots included in each shot family.

Shot family	Included shots
Smash	Powerful smash, over the fence smash, over the back fence smash, fake smash
*Bandeja*	*Bandeja, vibora, gancho, rulo*
Volleys	Forehand volley, backhand volley
Groundstrokes	Forehand, backhand
Walls	Back wall forehand/backhand, side wall forehand/backhand, double wall forehand/ backhand, *bajada de pared*
Other	*Cadete*, willy, *contrapared*, other

#### Coding conventions

Binary variables were coded 0/1 as specified; multi-level factors used descriptive labels. For specific pressure contrasts (e.g., Break vs Regular), the regular baseline excluded all other important-point types (golden and tiebreak).

### Procedure

The players were informed by the coach that they would undergo pressure training. Pressure training refers to an intervention designed to assist athletes in performing under pressure by deliberately exposing them to stressors during training sessions [25–27]. In our study, players were recorded during the practice matches while their technical-tactical performance was evaluated by the head coach of the first ever professional padel team of Finland.

An observer, a PhD student in Sports Sciences and certified padel coach with over 10 years of experience, observed the matches live and recorded the study variables through an *ad hoc* instrument. At the end of the collection process, an intra-observer reliability analysis was performed to ensure the veracity of the data collected. The observer reanalyzed a random sample of 6 matches (matches were recorded) to ensure enough relevant data to represent 10–20% of the study sample [28]. The mean intra-observer reliability was 0.91, considered almost perfect [29]. In addition, another observer, a PhD in Sports Sciences, certified padel coach and with a large number of published scientific research related to the topic of study, also analyzed a random sample of 6 matches to calculate the average interobserver reliability, which was 0.85 [29].

### Statistical analysis

#### First aim

For primary contrasts (important vs. regular points):

– Software. Primary analyses were conducted in jamovi (v2.6.26) using GAMLj (v3.6.1) [30].
– Outcomes and predictors. Point-level outcomes were binary: lastshot effectiveness (winner [W], forced error [FE], unforced error [UE]), serving pair wins the point (SrvWin), and point won by the set-winning pair (SetWinPt) (coded 1 = event, 0 = not). Point importance was coded as important (1) vs regular (0), and also as a four-level factor (Regular, Break, Golden, Tiebreak) for complementary contrasts.
– Model family and link. For each outcome we fit a binomial generalized linear mixed model (GLMM) with logit link and a random intercept for match (cluster = match_id). No additional covariance structure for repeated measures was specified. GLMMs were estimated by maximum likelihood with Laplace approximation using lme4::glmer (via jamovi/GAMLj).
– Fixed- and random-effects reporting. For fixed effects, we report odds ratios (OR = exp [β]) with 95% Wald CIs and two-tailed pvalues (α = 0.05) based on Wald χ^2^ tests. For random effects, we report the between-match variance and the intraclass correlation ICC with 95% CIs.
– Marginal effects. We present model-adjusted probabilities as estimated marginal means (EMMs) on the response (probability) scale, and the differences in percentage points (Δpp) with 95% CIs from marginal-effects contrasts (delta-method SEs). Withinoutcome multiple comparisons for the three “point type vs. Regular” contrasts use Holm adjustment.
– Model adequacy and fit. We assessed overdispersion using Pearson χ^2^/df (values ≈1 indicate no overdispersion) and confirmed model convergence. Log-likelihood, AIC and BIC are reported in the tables being a better model when lower values are reported.
– Fallback to generalized linear model (GLM) where appropriate. When the random-effect variance was at (or near) the boundary (singular fit; ICC ≈ 0) or the model warned of a singular fit, we refit the model as a population-average binomial GLM (same fixed effects, no random effect), estimated with base R stats: glm by iteratively reweighted least squares. This occurred for SetWinPt.
– Missing data. There were no missing data.
– Power. No a priori power was conducted. All analyses were performed on the full set of available matches/points.
– Transparency. Reporting follows recommendations for transparent GLMM analyses in psychology [31].

For the additional contrasts (Break/Golden/Tiebreak vs Regular):

– We repeated the models using point type (Regular as reference) to obtain Break vs Regular, Golden vs Regular, and Tiebreak vs Regular contrasts for each outcome. Because the random-effect variance for SrvWin and SetWinPt was (near) zero with point type, these were reported as GLMs; the other outcomes remained GLMMs. Within-outcome p-values for the three contrasts were Holm-adjusted.

#### Second aim

An inferential analysis was performed to develop contingency tables, including the Chi-square (χ^2^) statistical test to obtain the association between variables. The strength of association between variables was also calculated, for which Cramer’s V coefficient (Vc) was used [32]. Crewson (2006) differentiates the strength of association according to the value, considering a small (< 0.100), low (0.100–0.299), moderate (0.300–0.499) or high (> 0.500) association. In addition, subsequent Z-tests were performed to compare column proportions, adjusting for p values < 0.05 according to Bonferroni [33]. Contingency tables allowed identification of associations between variable categories through adjusted standard residuals (ASR). Residuals > |1.96| betrayed cells with more or fewer cases than there should be [32]. The significance level was set at p < 0.05 and statistical analysis was performed using the IBM SPSS (IBM SPSS Statistics for Windows, Version 27.0. IBM, Armonk, NY, USA) statistical package.

#### Graphs

All the figures were made with Microsoft Excel for Windows (Version 2021, Microsoft Corp.., Redmond, WA, USA), and Python v3.0 (Python Software Foundation, Wilmington, DE, USA).

## RESULTS

### Important points (all combined) vs. regular points

The GLMMs for UE, FE, W, and SrvWin converged normally and showed no overdispersion (Pearson χ^2^/df 0.994–1.000). The GLMM for SetWinPt reached a boundary (singular) random-effect variance (ICC ≈ 0%), so we report a population-average binomial GLM for that outcome; this GLM also showed no overdispersion (Pearson χ^2^/ df = 1.00).

**FIG. 1:**
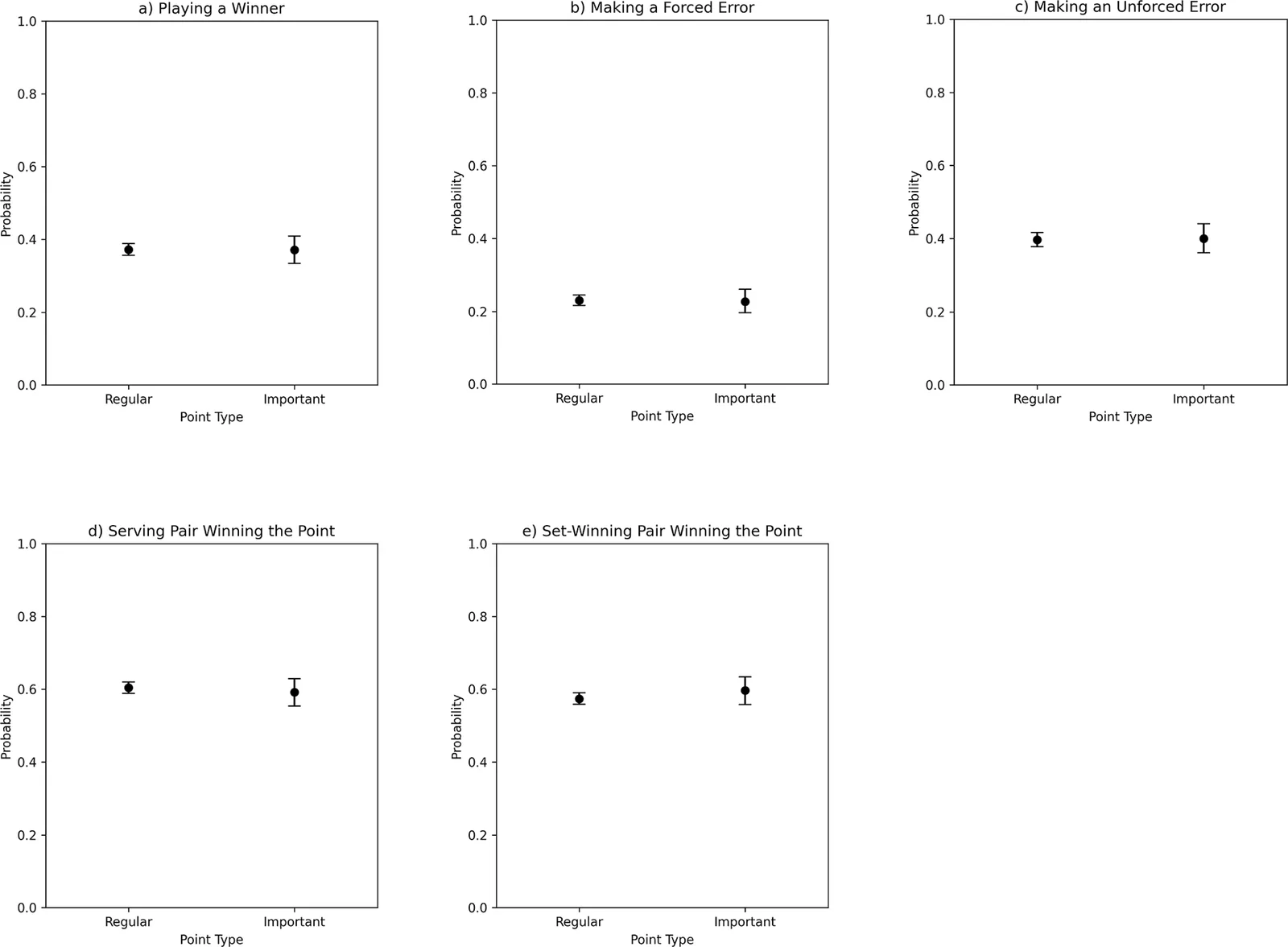
Model-adjusted probabilities (± 95% CI) on regular versus important points for each outcome: (a) winner, (b) forced error, (c) unforced error, (d) serving pair wins the point, (e) point won by set winners. Panels (a–d) display population-average predictions from binomial GLMMs with a random intercept for match (random effects not displayed). Panel (e) displays predictions from a binomial GLM (the GLMM’s random-effect variance was at the boundary). “Regular” denotes points that are not break, golden, or tiebreak.

Across outcomes, important vs. regular points showed no meaningful differences (Table 2; Figure 1): odds ratios were ~1.00 (range 0.95–1.10, all p ≥ 0.289), and model-adjusted probabilities differed by at most 2.3 percentage points (SetWinPt: 57.4% vs 59.7%); all other absolute differences were ≤ 1.2 pp.

**TABLE 2:** Important vs. regular points: odds ratios and model-adjusted probabilities.

Variable	Contrast	OR (95% CI)	Δ pp (95% CI)	p	Pred. prob. Regular, %	Pred. prob. Important, %	Var RE (match)	ICC %	Pearson χ^2^/df	LogLik	AIC / BIC
W	Important vs. Regular	1.00 (0.84–1.18)	−0.11 (-4.13−3.92)	0.958	37.2	37.1	0.00391	0.12	0.999	−2983.372	5972.744 / 5991.993

FE	Important vs. Regular	0.98 (0.81–1.20)	−0.32 (-3.82–3.18)	0.859	23.0	22.7	0.00733	0.22	0.997	−2440.436	4886.871 / 4906.121

UE	Important vs. Regular	1.01 (0.86–1.20)	+0.33 (-3.76–4.42)	0.875	39.7	40.0	0.02000	0.61	0.994	−3035.993	6077.986 / 6097.235

SrvWin	Important vs. Regular	0.95 (0.80–1.13)	−1.23 (-5.32–2.86)	0.555	60.4	59.2	0.00042	0.01	1.000	−3037.826	6081.653 / 6100.902

SetWinPt	Important vs. Regular	1.10 (0.93–1.30)	+2.23 (-1.86–6.31)	0.289	57.4	59.7		0.00	1.000	−3078.590	6161.17 / 6174.01

Note. Binomial models with logit link. UE/FE/W/SrvWin: generalized linear mixed models (GLMMs) with a random intercept for match; SetWinPt: binomial generalized linear model (GLM) because the GLMM random-effect variance was at the boundary (ICC ≈ 0%). “Regular” denotes points that are not break, golden, or tiebreak points. Predicted probabilities are model-adjusted estimated marginal means (response scale). Two-tailed α = 0.05. OR > 1 indicates higher odds on important vs. regular points. Abbreviations: Δpp: difference in percentage points; Var RE: random-effect variance; df: degrees of freedom; LogLik: log-likelihood; AIC: Akaike information criterion; BIC: Bayesian information criterion; UE: unforced error; FE: forced error; W: winner; SrvWin: serving pair wins the point; SetWinPt: set-winning pair wins the point.

### Important point types (break points, golden points, tiebreak points) vs. regular points

The GLMMs for UE, FE, and W converged with no overdispersion (Pearson χ^2^/df 0.995–0.999). For SrvWin and SetWinPt, the GLMMs exhibited boundary (singular) random-effect variance (ICC ≈ 0%); therefore, we report population-average binomial GLMs, each with no overdispersion (Pearson χ^2^/df = 1.00).

**FIG. 2:**
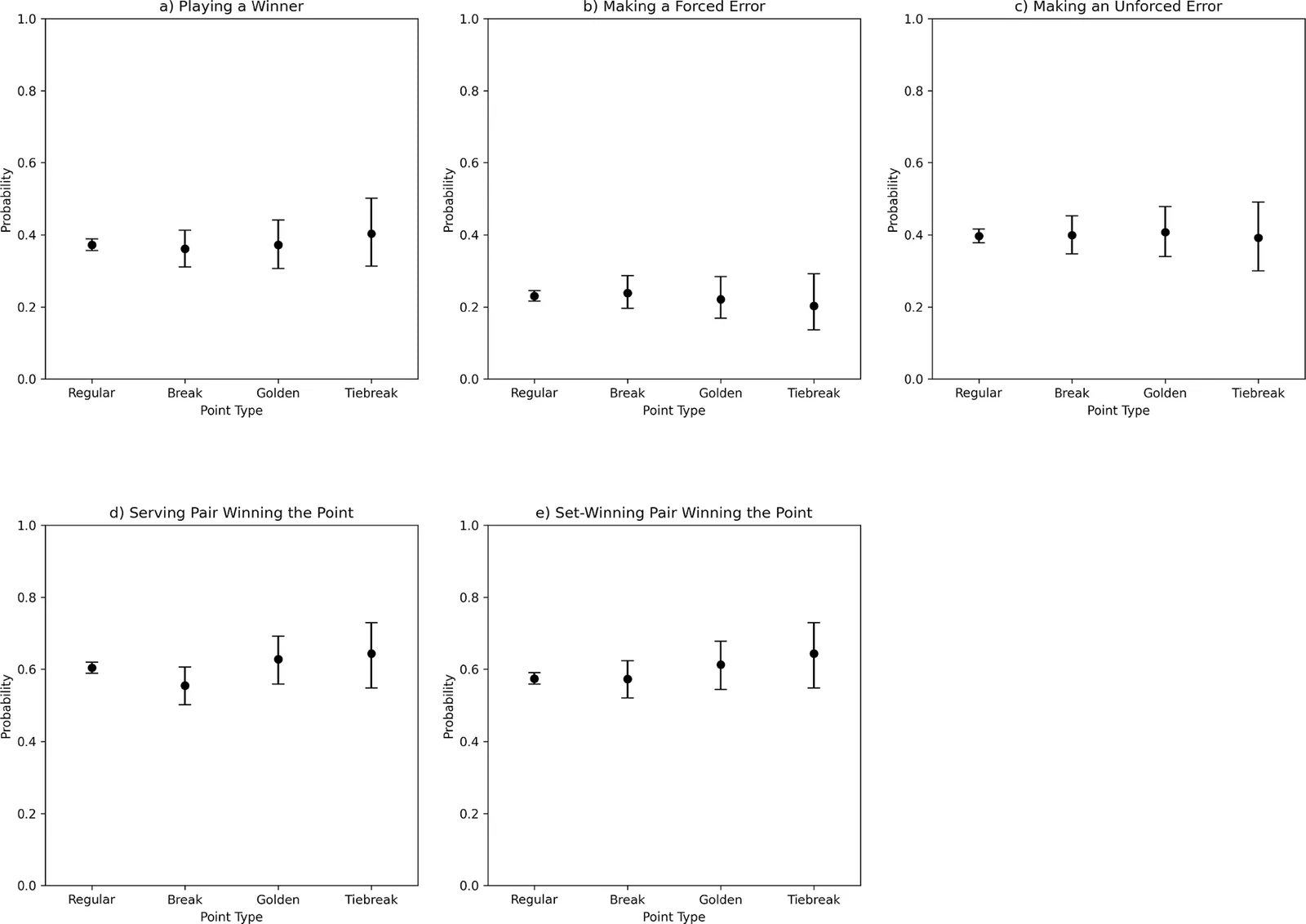
Model-adjusted probability (± 95% CI) by point type—Regular, Break, Golden, Tiebreak—for each outcome: (a) winner, (b) forced error, (c) unforced error, (d) serving pair wins the point, (e) point won by set winners. Panels (a–c) use binomial GLMMs with a random intercept for match (population-average predictions; random effects not displayed). Panels (d–e) use binomial GLMs (the corresponding GLMMs had boundary random-effect variance). “Regular” excludes break, golden, and tiebreak points.

Omnibus tests were non-significant for all outcomes (UE, FE, W: GLMMs; SrvWin, SetWinPt: GLMs; p = 0.209–0.992). Pairwise contrasts vs. regular were also non-significant after Holm adjustment (Table 3; Figure 2). Model-adjusted probability differences were modest: UE −0.5 to +1.0 pp; FE −2.7 to +0.8 pp; W −1.1 to +3.1 pp; SrvWin −4.9 to +4.0 pp; and SetWinPt −0.1 to +7.0 pp (CIs widely overlapping).

**TABLE 3:** Point type contrasts vs. regular points: odds ratios and model-adjusted probabilities.

Outcome	Contrast	OR (95% CI)	Δ pp (95% CI)	p	Pred. prob. Regular, %	Pred. prob. Contrast, %	Var RE (match)	ICC %	Pearson χ^2^/df	LogLik	AIC / BIC
W	BP vs. Regular	0.95 (0.76–1.20)	−1.13 (–6.43–4.18)	0.679	37.2	36.1	0.00358	0.11	0.999	–2983.070	5976.140 / 6008.223

	GP vs. Regular	1.00 (0.74–1.34)	0.00 (–6.91–6.87)	0.996	37.2	37.2	0.00358	0.11	0.999	–2983.070	5976.140 / 6008.223

	TB vs. Regular	1.14 (0.76–1.70)	+3.10 (–6.50–12.71)	0.520	37.2	40.3	0.00358	0.11	0.999	–2983.070	5976.140 / 6008.223

FE	BP vs. Regular	1.04 (0.81–1.35)	+0.78 (–3.92–5.48)	0.742	23.0	23.8	0.00697	0.21	0.998	–2440.120	4890.240 / 4922.323

	GP vs. Regular	0.95 (0.67–1.33)	−0.99 (–6.90–4.93)	0.748	23.0	22.1	0.00697	0.21	0.998	–2440.120	4890.240 / 4922.323

	TB vs. Regular	0.85 (0.52–1.39)	−2.74 (–10.67–5.20)	0.518	23.0	20.3	0.00697	0.21	0.998	–2440.120	4890.240 / 4922.323

UE	BP vs. Regular	1.01 (0.80–1.26)	+0.19 (–5.22–5.60)	0.946	39.7	39.9	0.02010	0.61	0.995	–3035.957	6081.913 / 6113.996

	GP vs. Regular	1.04 (0.78–1.40)	+1.01 (–5.99–8.02)	0.776	39.7	40.7	0.02010	0.61	0.995	–3035.957	6081.913 / 6113.996

	TB vs. Regular	0.98 (0.65–1.47)	−0.54 (–10.23–9.16)	0.914	39.7	39.2	0.02010	0.61	0.995	–3035.957	6081.913 / 6113.996

SrvWin	BP vs. Regular	0.82 (0.66–1.02)	−4.91 (–10.38–0.57)	0.075	60.4	55.5		0.00	1.000	–3035.730 6079.47 / 6105.13	

	GP vs. Regular	1.11 (0.82–1.49)	+2.39 (–4.50–9.28)	0.502	60.4	62.8		0.00	1.000	–3035.730 6079.47 / 6105.13	

	TB vs. Regular	1.19 (0.79–1.78	+3.99 (–5.33–13.32)	0.411	60.4	64.4		0.00	1.000	–3035.730 6079.47 / 6105.13	

SetWinPt	BP vs. Regular	0.99 (0.80–1.24)	–0.17 (–5.62–5.29)	0.952	57.4	57.3		0.00	1.000	–3077.570 6163.14 / 6188.81	

	GP vs. Regular	1.17 (0.88–1.57)	+3.87 (–3.07–10.82)	.281	57.4	61.3		0.00	1.000	–3077.570 6163.14 / 6188.81	

	TB vs. Regular	1.34 (0.89–2.02)	+6.99 (–2.34–16.32)	.156	57.4	64.4		0.00	1.000	–3077.570 6163.14 / 6188.81	

Note. Binomial models with logit link. UE/FE/W: generalized linear mixed models (GLMMs) with a random intercept for match; SrvWin and SetWinPt: binomial generalized linear model (GLM) because the GLMM random-effect variance was at the boundary (ICC ≈ 0%). “Regular” denotes points that are not break, golden, or tiebreak points. Each contrast compares Break (BP), Golden (GP), or Tiebreak (TB) to Regular. Predicted probabilities are model-adjusted estimated marginal means (response scale). p-values for the three contrasts within each outcome were adjusted by Holm’s method (inference unchanged vs unadjusted). Two-tailed α = 0.05. OR > 1 indicates higher odds in the contrasted point type vs. regular points. Abbreviations: Δpp: difference in percentage points; Var RE: random-effect variance; df: degrees of freedom; LogLik: log-likelihood; AIC: Akaike information criterion; BIC: Bayesian information criterion; BP: break point; GP: golden point; TB: tiebreak point; UE: unforced error; FE: forced error; W: winner; SrvWin: serving pair wins the point; SetWinPt: set-winning pair wins the point.

### Shot family composition in each kind of final shot effectiveness on important vs. regular points

**FIG. 3:**
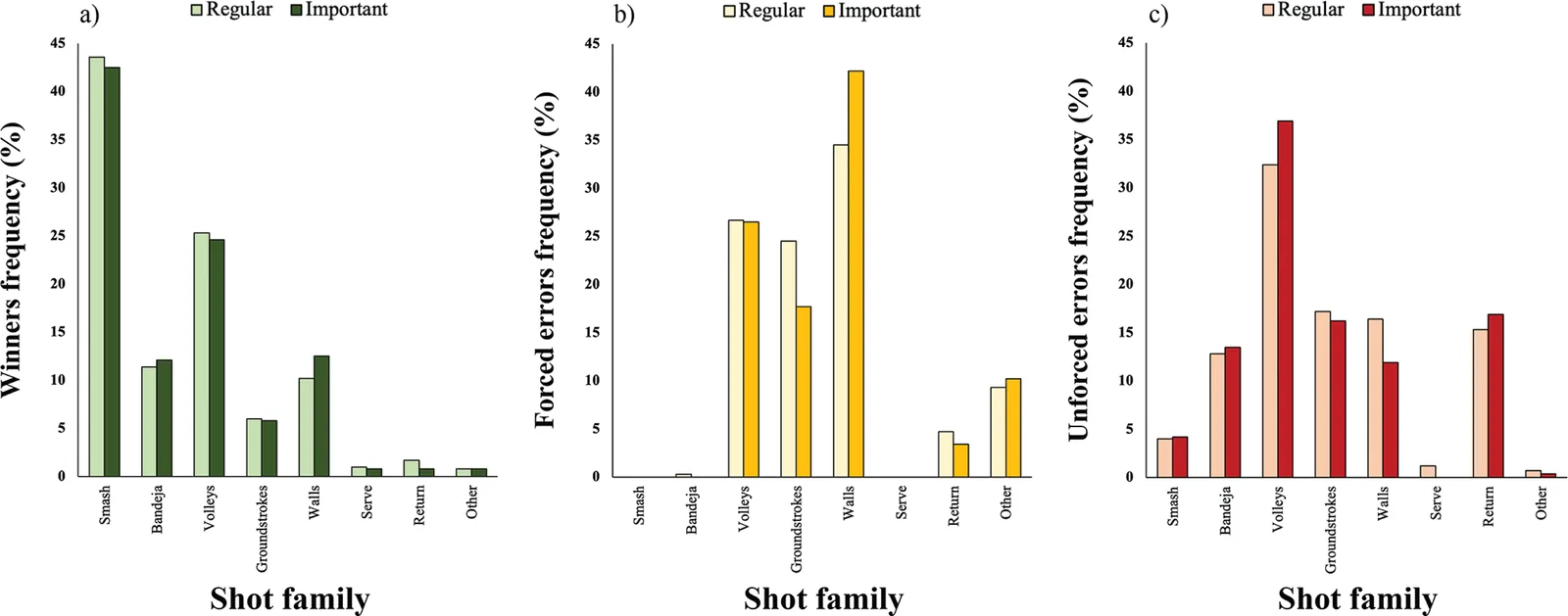
Shot family composition for winners (panel a), forced errors (panel b), and unforced errors (panel c). Darker bars represent important points, while lighter bars represent regular points.

The results showed no significant relationship between the type of last shot family and the importance of the point (important or regular) when the last shot was a winner (χ^2^(7) = 2.237; *p* = 0.946; *Vc* = 0.036), a forced error (χ^2^(5) = 5.624; *p* = 0.345.; *Vc* = 0.073), and an unforced error (χ^2^(7) = 8.229; *p* = 0.313; *Vc* = 0.068) (Table 4, Figure 3).

**TABLE 4:** Shot family composition in each kind of final shot effectiveness (W, FE, UE) on important vs. regular points.

Shot family	Winners						Forced errors						Unforced errors					

	Important			Regular			Important			Regular			Important			Regular		

	n	%	ASR	n	%	ASR	n	%	ASR	n	%	ASR	n	%	ASR	n	%	ASR
Smash	102	42.5a	−0.3	628	43.6a	0.3							11	4.2a	0.2	61	4.0a	−0.2
*Bandeja*	29	12.1a	0.3	164	11.4a	−0.3	0	0.0a	−0.7	3	0.3a	0.7	35	13.5a	0.3	197	12.8a	−0.3
Volleys	59	24.6a	−0.2	364	25.3a	0.2	39	26.5a	0.0	239	26.7a	0.0	96	36.9a	1.4	498	32.4a	−1.4
Groundstrokes	14	5.8a	−0.1	87	6.0a	0.1	26	17.7a	−1.8	219	24.5a	1.8	42	16.2a	−0.4	265	17.2a	0.4
Walls	30	12.5a	1.1	147	10.2a	−1.1	62	42.2a	1.8	309	34.5a	−1.8	31	11.9a	−1.8	252	16.4a	1.8
Serve	2	0.8a	−0.3	15	1.0a	0.3							0	0.0a	−1.8	18	1.2a	1.8
Return	2	0.8a	−1.0	24	1.7a	1.0	5	3.4a	−0.7	42	4.7a	0.7	44	16.9a	0.6	236	15.3a	−0.6
Other	2	0.8a	−0.0	12	0.8a	0.0	15	10.2a	0.4	83	9.3a	−0.4	1	0.4a	−0.6	11	0.7a	0.6

Note. n = number; % = percentage; ASR = adjusted standard residuals; ASR > 1.96: bold; a, b = indicate significant differences in the Z tests for comparison of column proportions from p < 0.05 adjusted according to Bonferroni.

## DISCUSSION

This study investigated whether technical-tactical performance at the point level differs between important points—defined as break points, golden points, and tiebreak points—and regular points in high-level men’s padel. This investigation addresses a significant gap in padel research, as understanding performance across different point types is essential for players and coaches. Contrary to our expectations, the results showed remarkable stability across contexts: the likelihood of winners, forced errors, unforced errors, the serving pair winning the points, and the set-winning pair winning the point did not differ meaningfully between important and regular points. Similarly, the distribution of shot families in the final shot of each rally in each of the last shot effectiveness options was unaffected by point importance.

Our first hypothesis suggested that the probability of making a winner, a forced error, an unforced error, the serving pair winning the point, and the set-winning pair winning the point would differ between regular and important points, with differences potentially varying across break, golden, and tiebreak contexts. This hypothesis was not supported at all (odds ratios consistently close to 1.00, non-significant omnibus tests, and modest probability differences ≤ ~3 percentage points). These findings suggest that highlevel male padel players from Finland maintain stable technical-tactical performance regardless of point importance. A likely explanation is that experienced players in this cohort have developed the psychological resilience and tactical consistency required to withstand high-pressure contexts. The nature of padel itself may also buffer against performance variation: unlike singles tennis, which is strongly influenced by serve dominance [34], padel involves longer rallies [35, 36], frequent defensive plays using the walls [37], and constant cooperation between partners [38]. Such features may reduce the weight of single actions and dilute pressure effects.

From a psychological perspective, outcome interdependence [39], and the predictability of tactical patterns [37, 40] may all contribute to maintaining stable performance in high-pressure moments. Together, these sport-specific and mental factors likely buffer the influence of pressure in padel, supporting the observed stability of technical–tactical indicators across contexts.

In addition, previous research in padel showed that winning pairs win more break points and golden points than losing pairs [9]. Notably, our findings differ from previous tennis research, where pressure has been shown to increase unforced errors [20], alter serving effectiveness [41], and favor the eventual match winners in converting break points [42]. Instead, our findings align with other sports literature suggesting that highly trained athletes preserve their performance under pressure [43]. Thus, high-level male padel players appear more resilient to situational pressure than might be expected from studies in other racquet sports.

In addition, although no differences emerged between important and regular points, the overall proportion of unforced errors was relatively high (~40%). These values exceed those reported for professional male players competing in qualification (~38%) and main draw matches (~31%) in the World Padel Tour [44]. Considering that padel is a relatively new sport in the country of the players analysed, and consistent with recent findings in professional padel [45], it is plausible that the frequency of unforced errors among this population of high-level male players will decrease as technical and tactical expertise continue to develop.

Our second hypothesis proposed that specific shots families would be more error-prone (e.g., smashes, *bandejas*, returns) or more effective (e.g., volleys) in important points compared to regular points. This hypothesis was also not accepted. The composition of shot families in the last stroke did not vary significantly between important and regular points across winners, forced errors, and unforced errors. This reinforces the notion that high-level padel players’ technical-tactical actions at the rally end remain robust in decisive contexts. A possible explanation is that high-level male players from Finland rely on well-rehearsed tactical repertoires that they execute consistently regardless of point importance, rather than altering their final-shot selection in high-stakes moments. Nevertheless, research in tennis [46] and in experimental motor-control tasks [47, 48] shows that pressure can shift decision making toward safer choices or elicit ironic performance effects, where attempts to avoid specific errors paradoxically increase their likelihood. In controlled tasks, avoidant instructions under pressure (e.g., “don’t hit here”) increase targetzone mistakes, and pressure can modulate the balance between ironic and over-compensation errors [48, 49]. These mechanisms offer plausible pathways for context-dependent adjustments even if our aggregated final-shot categories did not change.

### Strengths, limitations and future research directions

A major strength of this investigation is that it represents the first study to systematically compare important versus regular points in high-level padel, providing novel insights into how match context influences technical–tactical performance. Another strength lies in the rigorous analytical approach: the use of generalized linear mixed models (GLMMs) allowed us to account for repeated measures within matches and to minimize statistical bias.

Nevertheless, several limitations should be acknowledged. First, the sample was restricted to men’s padel in Finland, which limits the generalizability of the findings to other populations, competitive levels, and playing styles. Second, women’s and youth categories were not included, leaving unanswered questions about whether pressure effects differ across sex or developmental stages. Third, the analysis focused exclusively on observable technical–tactical outcomes, without incorporating psychological or physiological indicators of pressure (e.g., perceived stress, heart rate variability), which may provide complementary explanations of performance under highstakes conditions.

Future studies should address these limitations by extending analyses to professional circuits, female and youth competitions, and by integrating multimodal data (technical-tactical, psychological, and physiological). Incorporating a continuous pressure index—based on point importance within the match—may also provide a more nuanced measure of situational stress than categorical important versus regular points. Finally, differentiating between winning and losing pairs during important points could help identify subtler adaptations and distinguish “clutch” from “non-clutch” performance profiles.

*Practical implications*

The present findings carry important implications for understanding performance in padel. From a theoretical perspective, they suggest that the concept of choking under pressure, defined as a breakdown in performance under conditions of high importance [50], may be less applicable to high-level padel players, at least at the aggregated level of observable technical–tactical outcomes. Instead, padel may exemplify a sport where expertise, cooperative dynamics, and contextual features (e.g., extended rallies) collectively mitigate the negative effects of pressure. From a practical perspective, these results encourage coaches to focus on reinforcing consistency and resilience, rather than expecting drastic tactical shifts in important points. Pressure-training tasks, such as practicing exclusively with goldenpoint scoring or starting games at 30–40, may still be valuable, not because players collapse under pressure, but because rehearsing these scenarios may help consolidate the routines and communication strategies that underpin stable execution. Mental skills training should continue to be emphasized, not as a remedy for breakdowns, but as a reinforcement of effective routines, arousal regulation, and attentional control that already appear to function well in competitive contexts. In doubles settings like padel, specific attention to pair communication and role clarity may also be key, as shared responsibility likely contributes to stability in high-stakes moments.

## CONCLUSIONS

In high-level men’s padel from Finland, point importance was not associated with systematic differences in last-shot effectiveness (winners, forced/unforced errors), serving pair success, set-winning pair success, or final-shot family. Technical–tactical patterns at point end were stable across important and regular points. Practically, training may prioritize consistent execution across contexts; pressure drills can rehearse decisive scenarios without presuming changes in finalshot selection or effectiveness.

## Data Availability

Data will be available upon request.
